# Assessment of Enhancement Kinetics Improves the Specificity of Abbreviated Breast MRI: Performance in an Enriched Cohort

**DOI:** 10.3390/diagnostics13010136

**Published:** 2022-12-30

**Authors:** Haejung Kim, Eun Young Ko, Ka Eun Kim, Myoung Kyoung Kim, Ji Soo Choi, Eun Sook Ko, Boo-Kyung Han

**Affiliations:** Department of Radiology and Center for Imaging Science, Samsung Medical Center, Sungkyunkwan University School of Medicine, Seoul 06351, Republic of Korea; Hjk220@naver.com (H.K.); ganne1113@gmail.com (K.E.K.); myoungkyoung.kim@samsung.com (M.K.K.); jisoo.choi@samsung.com (J.S.C.); es.ko@samsung.com (E.S.K.); bkhan@skku.edu (B.-K.H.)

**Keywords:** abbreviated breast MRI, breast, breast cancer, kinetics, MRI

## Abstract

Objective: To investigate the added value of kinetic information for breast lesion evaluation on abbreviated breast MRI (AB-MRI). Methods: This retrospective study analyzed 207 breast lesions with Breast Imaging Reporting and Data System categories 3, 4, or 5 on AB-MRI in 198 consecutive patients who had breast MRI for screening after breast cancer surgery between January 2017 and December 2019. All lesions were pathologically confirmed or stable on follow-up images for 2 years or more. Kinetic information of the lesions regarding the degree and rate of enhancement on the first post-contrast-enhanced image and the enhancement curve type from two post-contrast-enhanced images were analyzed on a commercially available computer-assisted diagnosis system. The diagnostic performances of AB-MRI with morphological analysis alone and with the addition of kinetic information were compared using the McNemar test. Results: Of 207 lesions, 59 (28.5%) were malignant and 148 (71.5%) were benign. The addition of an enhancement degree of ≥90% to the morphological analysis significantly increased the specificity of AB-MRI (29.7% vs. 52.7%, *p* < 0.001) without significantly reducing the sensitivity (94.9% vs. 89.8%, *p* = 0.083) compared to morphological analysis alone. Unnecessary biopsy could have been avoided in 34 benign lesions, although three malignant lesions could have been missed. For detecting invasive cancer, adding an enhancement degree ≥107% to the morphological analysis significantly increased the specificity (26.5% vs. 57.6%, *p* < 0.001) without significantly decreasing the sensitivity (94.6% vs. 86.5%, *p* = 0.083). Conclusion: Adding the degree of enhancement on the first post-contrast-enhanced image to the morphological analysis resulted in higher AB-MRI specificity without compromising its sensitivity.

## 1. Introduction

Breast MRI is the most sensitive imaging modality for detecting breast cancer [1,2,3]. However, high costs and long scan and interpretation times are major drawbacks for the wide availability of breast MRI as a screening method [4]. To increase the availability of MRI, Fischer et al. [5] initially presented a new concept of first-pass MRI, which consists of two post-contrast sequences with a total examination time of 4–5 min in the case of normal findings. Subsequently, Kuhl et al. [6] proposed the concept of abbreviated MRI (AB-MRI), which consists of one pre- and one post-contrast acquisition and their derived images (first post-contrast-subtracted and maximum-intensity projection [MIP]). Following these initial studies, multiple studies have reported that the diagnostic accuracy of AB-MRI is equivalent to that of the conventional full diagnostic protocol of MRI (FDP-MRI), with shorter acquisition and reading times, thereby making it more cost-effective as a screening tool [7,8,9,10,11,12,13,14,15,16].

Although morphological analysis is the first step of breast MRI interpretation, kinetic information helps differentiating benign from malignant lesions [17,18,19]. Enhancement kinetics analysis of FDP-MRI is performed at two different phases: early phase enhancement describing the steepness of the first part of the kinetic curve, during the first 1-2 min after contrast injection and delayed phase enhancement describing the time–signal intensity curve, at 3 min or more after contrast injection [19]. Consequently, not acquiring delayed contrast-enhanced images on AB-MRI does not mean that kinetic information is not provided. AB-MRI obtains the most important early kinetic information, which is a key component of AB-MRI. As cancers enhance faster and stronger than normal tissue or benign lesions, early post-contrast imaging is best for detecting invasive breast cancer (IDC) and high-grade ductal carcinoma in situ (DCIS) [6,10,20]. Similarly, as the contrast between cancer and background parenchymal enhancement (BPE) is the highest during the early post-contrast phase, morphological characteristics could also be obtained more easily during this early phase [21,22,23]. Although identifying significant enhancements (i.e., enhancement beyond physiologic BPE) in the MIP images is the first step in interpreting AB-MRI based on the original concept, little has been reported regarding the added value of this early kinetic information to morphological analysis alone for differentiating benign from malignant breast lesions on AB-MRI.

In 2018, Comstock and Kuhl [24] suggested a standardized method for AB-MRI interpretation, which recommended whether a follow-up or biopsy should be performed based on the morphological features of the lesions. Therefore, the purpose of this study was to investigate whether adding kinetic information to morphological analysis improves the diagnostic performance of AB-MRI by using the interpretation guideline, and to suggest the best way to use the abbreviated protocol of MRI for breast cancer screening.

## 2. Materials and Methods

### 2.1. Patients

**Figure 1:**
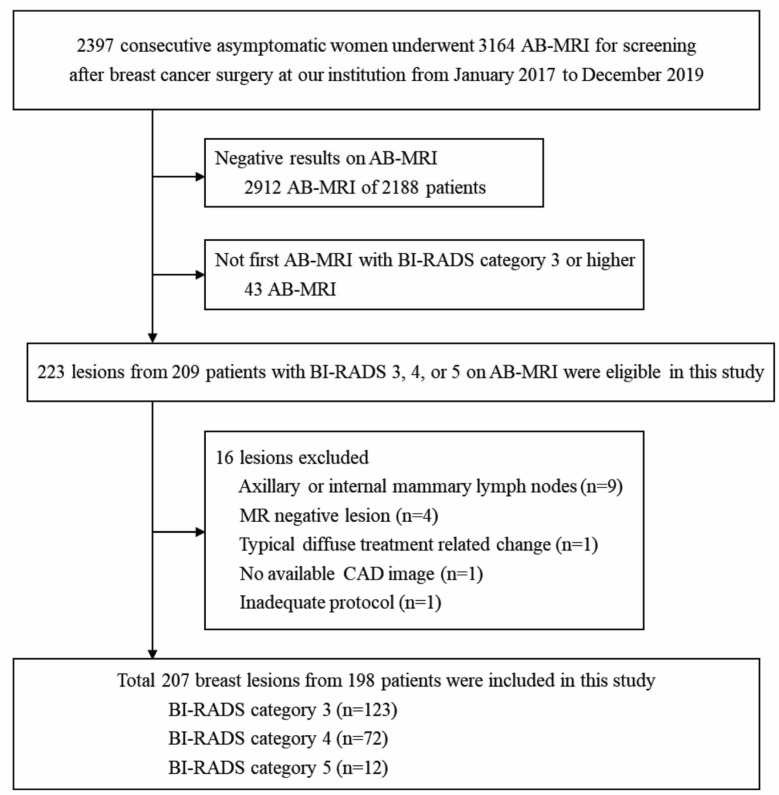
Flowchart of the study population and exclusion criteria. AB-MRI = abbreviated breast MRI, BI-RADS = Breast Imaging Reporting and Data System, CAD = computer-assisted diagnosis.

This retrospective study was approved by the Institutional Review Board of the Ethics Committee, which waived the requirement for informed consent. Our institution has used breast AB-MRI to screen women at all risk for breast cancer since September 2015, but most of them had a personal history of breast cancer. Postoperative screening MRI has not been routinely performed for all patients with a personal history of breast cancer. Some had dense breast, were diagnosed with breast cancer at <50 years of age, had high-risk factors (breast cancer gene (*BRCA*) mutation, family history of breast cancer), or had a history of interstitial mammoplasty, and underwent postoperative screening MRI. This was additionally performed at the request of patients or clinicians. From January 2017 to December 2019, a total of 2397 asymptomatic women with a personal history of breast cancer underwent 3164 consecutive AB-MRI scans to screen for breast cancer after surgery. Among them, 2912 AB-MRI of 2188 patients with Breast Imaging Reporting and Data System (BI-RADS) categories 1 or 2 were excluded. As we included only one AB-MRI per patient, which was the first AB-MRI with BI-RADS category 3 or higher during the period; an additional 43 follow-up AB-MRI were excluded. Finally, 209 AB-MRI of 209 patients with BI-RADS categories 3, 4, or 5 were considered eligible for our study. Of 209 patients, 14 had two lesions with BI-RADS category 3 or higher per AB-MRI. Among 223 lesions of 209 patients, 16 (nine extramammary lymph nodes, four MR negative lesions with final assessment based on their integration with other modalities, one typical diffuse treatment-related change, one with no available computer-assisted diagnosis (CAD) image, and one inadequate protocol) lesions were excluded. Finally, 207 lesions from 198 patients were included (Figure 1). All lesions were confirmed as benign or malignant by biopsy or were considered benign when the lesion showed benign findings and stability on follow-up imaging studies for at least 2 years. Of the 84 lesions that were confirmed by biopsy, 80 lesions showed corresponding lesions on US and had US-guided biopsy, one lesion showed suspicious calcifications in corresponding area and underwent stereotactic biopsy, and three lesions had MR-guided biopsy. Of the 198 patients, nine had two lesions with BI-RADS category 3 or higher; two had two benign lesions, four with one benign and one malignant lesion, and three had two malignant lesions. The results of other imaging studies, including ultrasound and mammography, were not considered. Of the 198 AB-MRI, 136 were the first screening round, while 62 were the subsequent screening round. The median interval between cancer surgery and the screening AB-MRI was 21.1 months (interquartile range, 13.8–31.9 months). The pathology and electronic medical records were reviewed.

### 2.2. Breast MRI Acquisition

All AB-MRI scans were performed using a 3.0- or 1.5-T Achieva scanner (Philips Medical Systems) with a dedicated bilateral phased-array breast coil. The protocol included a T2-weighted sequence, one pre-contrast sequence, and two post-contrast-enhanced sequences. The contrast-enhanced images were obtained after a 0.1-mmol/kg bolus injection of gadobutrol (Gadovist; Bayer Healthcare, Berlin, Germany), followed by a 20-mL saline flush. Scanning for post-contrast imaging began 30 s after the contrast injection, with a temporal resolution of 60 s. The first post-contrast sequence was obtained from 30 s to 90 s after the beginning of the scan, and the second post-contrast sequence was sequentially obtained from 90 s to 150 s. After image acquisition, the subtraction and bilateral axial and sagittal MIP images were reformatted. The parameters for a 3.0-T scanner were: repetition time (TR)/echo time (TE) (ms), 4.6/2.3; field of view, 32 × 32 cm; matrix size, 512 × 512; flip angle, 24°; 1.5 mm sections with no gap; those for a 1.5-T scanner were: TR/TE (ms), 6.5/2.5; field of view, 32 × 32 cm; matrix size, 376 × 374; flip angle, 12°; 1.5 mm sections with no gap. The total scan time was 10 min or less (Supplementary Table S1). 

### 2.3. Morphological Analysis

The findings of the lesions on AB-MRIs were retrospectively reviewed by two breast radiologists (E.Y.K. and H.K., with 19 and 2 years of breast MRI experience, respectively). Discordant cases were discussed to reach a consensus. Morphological analysis was performed according to the suggested interpretation guidelines [24] and diagnosed as positive when biopsy was recommended and negative when follow-up was recommended or benign findings were suggested (Supplementary Figures S1 and S2). The lesions were divided into mass and non-mass enhancement. The margins (circumscribed vs. irregular/spiculated), T2 signal intensities (high vs. not high), internal enhancement patterns (homogeneous vs. heterogeneous), and presence of rim enhancement were analyzed for the masses, whereas the distributions (linear/segmental vs. focal/regional/multiple regions/diffuse) and internal enhancement patterns (homogeneous vs. heterogeneous/clumped/clustered ring) were analyzed for non-mass enhancement. The size of the enhancing lesion was defined as the largest diameter. 

### 2.4. Kinetic Analysis

Kinetic information was retrospectively analyzed on a dedicated workstation using a CAD system (CadstreamTM v6.0, Merge Healthcare, Inc., Hartland, WI, USA). If pixel value increased above a user-defined minimum enhancement threshold on first post-contrast images compared to pre-contrast images, the pixel was automatically identified by color overlays on each MRI slice. We defined minimum enhancement threshold as 50% increase in enhancement. Kinetic analysis of the enhancing lesions detected by CAD was automatically performed by clicking on the lesion, without the need to directly draw regions of interest. The enhancement degree was defined as peak enhancement percentage on the first post-contrast images. The enhancement rate, defined as the signal change between the pre- and first post-contrast images, was categorized as slow (<50% increase), medium (50–100%), or rapid (>100%). The enhancement curve, determined by the signal change between the first and second post-contrast images, was categorized as persistent (>10% increase), plateau (≤10% increase or ≤10% decrease), or washout (>10% decrease). When two or more types of curve were mixed within the same lesion, the most suspicious curve type was recorded. For lesions that could not be detected by CAD, we manually identified a single voxel within each lesion in the area of the highest enhancement on the first post-contrast images. The kinetic information was automatically generated from this voxel. 

### 2.5. Statistical Analysis

Continuous variables, including age, tumor size, and enhancement degree, were analyzed using Student *t*- and Wilcoxon rank sum tests. Categorical variables including family history of breast cancer, BRCA mutation, MRI features, enhancement rate, enhancement curve type, BI-RADS category, biopsy recommend by interpretation guideline, MRI magnetic field strength, and screening round were compared using Fisher exact and *x*2 tests. The diagnostic performances of morphological analysis following the interpretation guideline, kinetic information including enhancement degree, enhancement rate, enhancement curve type, and tumor size were calculated by measuring the area under the receiver operating characteristic (ROC) curve (AUC), which were compared using the DeLong test. After identifying parameters with significantly higher AUC compared to morphologic analysis alone, we defined their optimal cutoff values as the points that increased the specificity of the guideline without significantly sacrificing its sensitivity to continuous variables and applied the Youden index for categorical variables. The diagnostic performances of morphological analysis alone and morphology with kinetic information were compared using the McNemar test. Two-sided *p* < 0.05 was considered statistically significant. All statistical analyses were performed using SAS software (version 9.4; SAS Institute, Cary, NC, USA).

## 3. Results 

### 3.1. Patient and Lesion Characteristics

The patient and lesion characteristics are summarized in Table 1. Of the 207 lesions, 59 (28.5%) were malignant (35 IDCs, 22 DCIS, and 2 metaplastic carcinomas) and 148 (71.5%) were benign (113 stable for ≥2 years on imaging follow-up; 10 fat necrosis; 8 fibroadenomas; 4 stromal fibrosis; 4 fibrocystic changes; 2 intraductal papillomas; 2 benign breast tissues; and 1 each of sclerosing adenosis, usual ductal hyperplasia, adenomyoepithelioma, cholesterol granuloma, and fibromatosis confirmed by biopsies). Both groups had more women without family history of breast cancer (88.1–91.2%) than women with family history of breast cancer (8.8–11.9%), and more women without BRCA mutation (81.4–87.8%) than with BRCA mutation (12.2–18.6%). The malignant lesions were significantly larger (*p* < 0.001). Mass was a more common lesion type in both benign and malignant lesions; however, non-mass enhancement was more common in malignant lesions than in benign lesions (30.5% vs. 17.6%, *p* = 0.040). In the morphological analysis, malignant masses more frequently showed non-circumscribed margins (*p* < 0.001), heterogeneous enhancement (*p* = 0.012), and rim enhancement (*p* = 0.014). Furthermore, non-mass lesions showed no significant differences between benign and malignant lesions in morphological analysis. In the kinetic analysis, malignant lesions showed a significantly higher degree of enhancement (*p* < 0.001) and more frequently showed rapid enhancement rates (*p* < 0.001) than the benign lesions. None of the malignant lesions showed slow enhancement rate. Regarding the enhancement curve type, malignant lesions were more likely to show washout type than benign lesions, whereas the latter were more likely to feature persistent lesions compared to malignant lesions (*p* < 0.001). Benign lesions were more likely to be assessed as BI-RADS category 3 than malignant lesions (77.0% vs. 15.3%, *p* < 0.001), while none of the benign lesions were assessed as BI-RADS category 4C and 5. According to the interpretation guideline, 70.3% of benign lesions were recommended to undergo biopsy while 94.9% malignant lesions were recommended for biopsy (*p* < 0.001). MRI magnetic field strength of 3.0-T (68.9–74.6%) were more frequent than 1.5-T (25.4–31.1%) for both groups, and most AB-MRI were the first screening round (66.2–71.2%).

### 3.2. Receiver Operating Characteristics Curve Analysis of Parameters for Differentiating Benign and Malignant Breast Lesions

**Figure 2:**
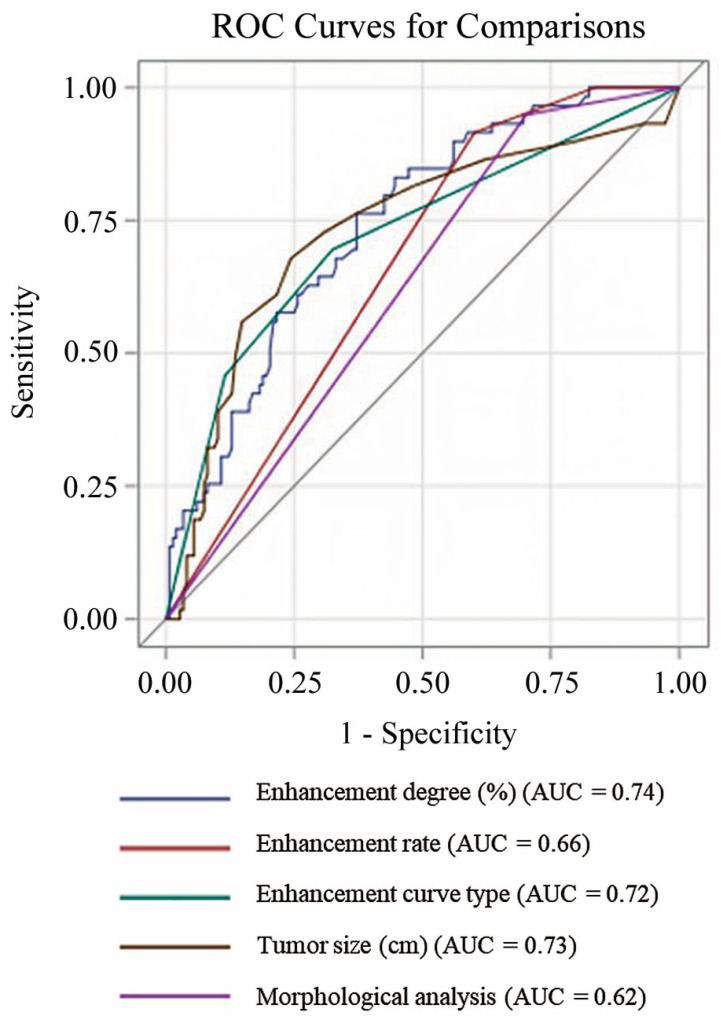
ROC curves of the parameters for differentiating malignant from benign breast lesions. ROC = receiver operating characteristic.

**Figure 3:**
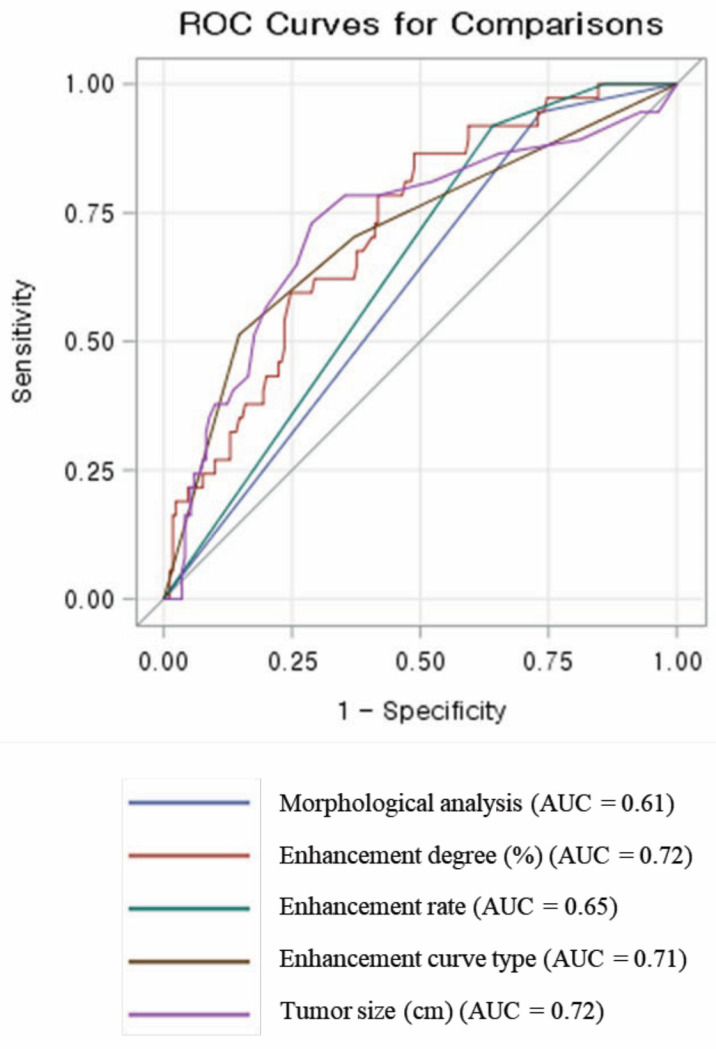
ROC curves of the parameters for detecting invasive cancer. ROC = receiver operating characteristic.

The AUC values of the morphological analysis following AB-MRI interpretation guidelines, kinetic parameters including enhancement degree, enhancement rate, enhancement curve type, and size are shown in Table 2. For the detection of all malignancies including in situ carcinoma, the enhancement degree, enhancement curve type, and size showed significantly better AUC values compared to morphological analysis alone (0.72–0.74 vs. 0.62; *p* < 0.05 for all parameters). The ROC curves for each parameter are shown in Figure 2. For the detection of invasive cancers, enhancement degree and size showed significantly better AUC values compared to morphological analysis alone (0.72 for each vs. 0.61; *p* < 0.05 for both parameters). The ROC curves for each parameter are shown in Figure 3.

### 3.3. Optimal Cutoff Values of the Parameters

As shown in Table 2, the optimal cutoff values of enhancement degree that showed the highest specificity without significantly decreasing sensitivity were 90% for the detection of all malignancy and 107% for the detection of invasive cancer. The cutoff value of the enhancement curve type for the detection of all malignancies was defined as a plateau by the Youden index, which was the point that maximized the sum of sensitivity and specificity. Although a tumor size of 0.5 cm was the limit that significantly improved thw specificity of the guidelines for the detection of all malignancies, we were unable to set the cutoff value because the sensitivity was significantly decreased. The tumor size of 0.6 cm was selected as the cutoff for the detection of invasive cancers. 

### 3.4. Diagnostic Performance of the Combined Morphology and Kinetic Information Compared to Morphological Analysis Alone

**Figure 4:**
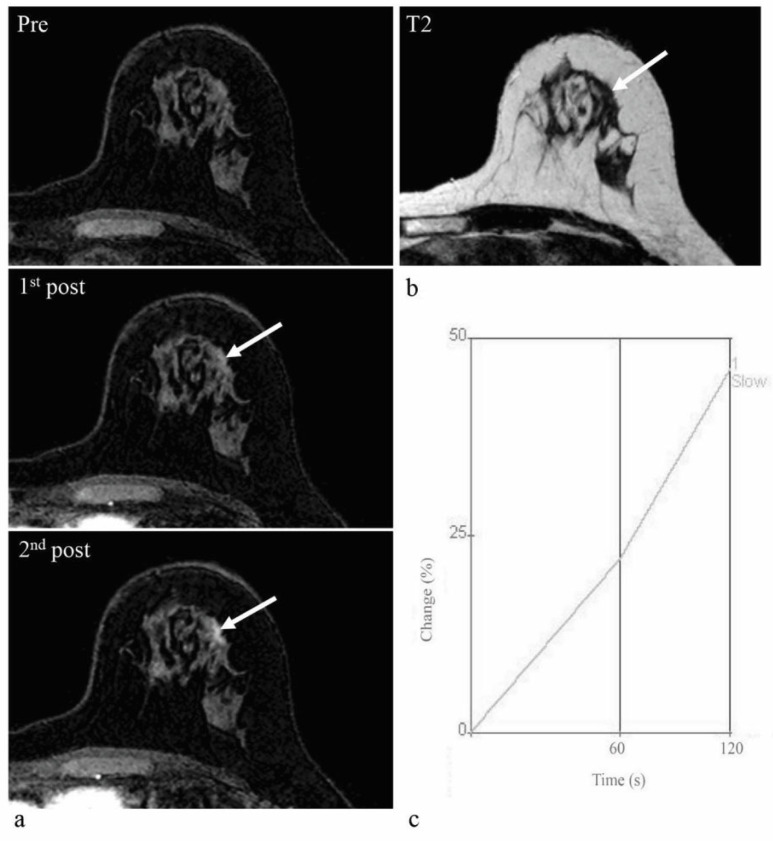
Screening abbreviated breast MRI (AB-MRI) images of a 48-year-old woman with a history of right total mastectomy and left breast-conserving surgery due to bilateral breast cancer. Two years after surgery, pre-contrast and two post-contrast-enhanced axial images (**a**) show a 0.9 cm irregular enhancing mass (arrows) in the left breast at the 1 o’clock direction. The T2 signal intensity is not high (**b**). This lesion was assessed as BI-RADS 4B category in the original report. According to the interpretation guideline, it is classified as a suspicious lesion for which biopsy is recommended. However, kinetic analysis shows a low enhancement degree of 22% on the first post-contrast-enhanced image (**c**). This lesion was detected by second-look ultrasound (US) and confirmed as sclerosing adenosis by US-guided core biopsy. In conclusion, considering kinetic information could have avoided an unnecessary biopsy of this false-positive lesion.

**Figure 5:**
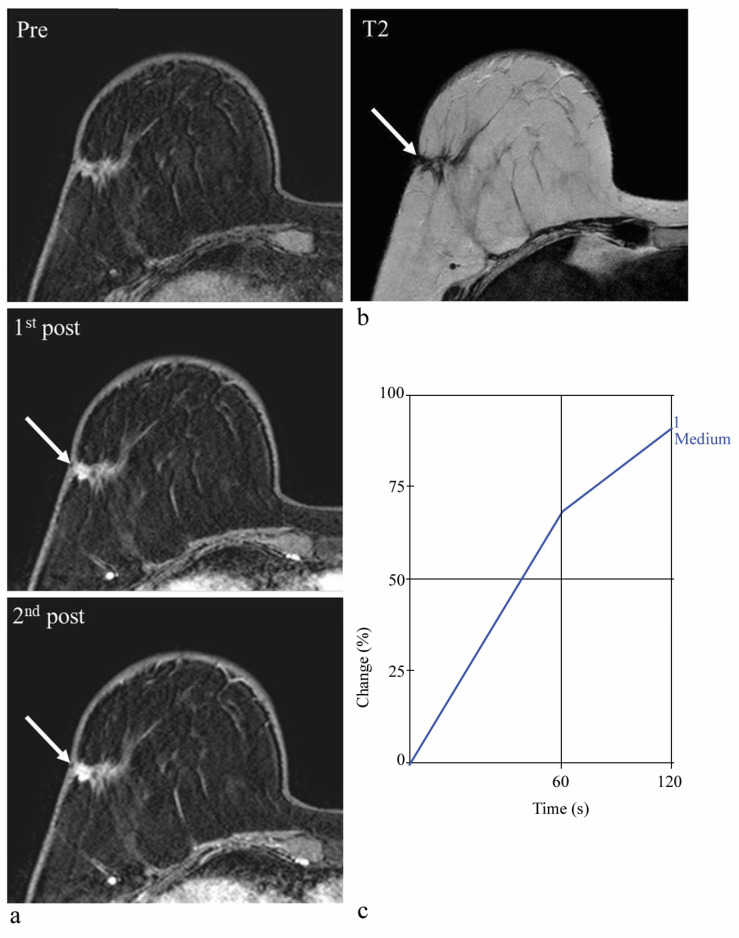
Screening abbreviated breast MRI (AB-MRI) images of a 67-year-old woman with a history of right breast-conserving surgery due to breast cancer. Four years after surgery, pre-contrast and two post-contrast-enhanced axial images (**a**) show a 1.1 cm irregular enhancing mass (arrows) in the right breast at the operation site. The T2 signal intensity is not high (**b**). This lesion was assessed as BI-RADS 4C category in the original report. According to the interpretation guideline, it is classified as a suspicious lesion for which biopsy is recommended. However, kinetic analysis shows a low enhancement degree of 68% on the first post-contrast-enhanced image (**c**). This lesion was detected by second-look ultrasound (US) and confirmed as invasive ductal carcinoma by US-guided core biopsy and subsequent surgery. This is a false-negative case when the enhancement degree on the first post-contrast-enhanced image is combined with morphological analysis.

The results of a comparison of the diagnostic performances are shown in Table 3. AB-MRI with morphological analysis alone, following the interpretation guidelines, showed 94.9% sensitivity and 29.7% specificity. When only lesions with enhancement degree ≥90% among the biopsy-recommended lesions according to the interpretation guidelines undergo biopsy, the specificity of AB-MRI significantly increassed (52.7%, *p* < 0.001) without significantly reducing sensitivity (89.8%, *p* = 0.080). Thus, 34 benign lesions for which biopsy was recommended by morphological analysis alone would be classified as negative after considering the degree of enhancement (Figure 4). However, three malignant lesions, one DCIS and two IDCs, could have been missed (Figure 5). A slow enhancement rate was observed only in benign lesions; however, as the AUC value of enhancement rate was not significantly higher than the guideline, the addition of the enhancement rate to the morphological analysis was not analyzed. When the enhancement curve type (plateau or washout) was added to the morphological analysis, the specificity significantly increased to 79.1% (*p* < 0.001), but the sensitivity also significantly decreased to 66.1% (*p* < 0.001). Adding both enhancement curve type (plateau or washout) and enhancement degree (≥90%) also significantly increased in specificity to 80.4% (*p* < 0.001), but significantly decreased in sensitivity to 64.4% (*p* < 0.001). 

For the detection of invasive cancer, AB-MRI following the interpretation guidelines showed 94.6% sensitivity and 26.5% specificity. When an enhancement degree of ≥107% was added to the morphological analysis, the specificity significantly increased (57.6%, *p* < 0.001), and the sensitivity was not significantly reduced (86.5%, *p* = 0.083). Adding size ≥0.6 cm to the morphological analysis also significantly increased the specificity (38.8%, *p* < 0.001) without a significant decrease in sensitivity (86.5%, *p* = 0.083). Adding both enhancement degree ≥107% and size ≥0.6 cm to the morphologic criteria significantly increased the specificity (63.5%, *p* < 0.001), but also significantly reduced the sensitivity (78.4%, *p* = 0.014). 

In the subgroup analysis, according to the magnetic field strengths (1.5-T vs. 3.0-T), adding an enhancement degree of ≥90% to detect all malignancy or ≥107% for invasive cancer to the morphological analysis significantly increased the specificity in both 1.5-T and 3.0-T (*p* < 0.001). The sensitivity decreased only at 1.5-T, but not significantly (*p* = 0.083). Adding size ≥0.6 cm to the morphologic criteria significantly increased the specificity (37.8%, *p* < 0.001) at 3.0-T, without a significant decrease in sensitivity (85.2%, *p* = 0.157).

Finally, the only parameter that significantly improved specificity without significantly compromising sensitivity was the degree of enhancement (≥90% for all malignancy; ≥107% for invasive cancer) at both magnetic field strengths.

## 4. Discussion

Our study demonstrated that the use of the quantitative enhancement degree on first post-contrast images significantly improved the specificity of AB-MRI in differentiating benign from malignant breast lesions. We also proposed cutoff values of 90% for detecting all malignancy and 107% for invasive cancer, which significantly increased the specificity without sacrificing the sensitivity of AB-MRI. These results were consistent for both 1.5-T and 3.0-T MRI. 

Kinetic information is a key component of the concept of AB-MRI, as identifying the significant enhancement on the MIP images is the first step in AB-MRI interpretation [6]. However, no evidence is available regarding the explicit use of kinetics in conjunction with the pure morphological assessment of AB-MRI. In practice, many factors may affect the final determination of the BI-RADS category, including radiologists’ experience, available previous imaging findings, patient history, and the subjective interpretation of kinetic information by visually assessing the dynamic images due to CAD system unavailability. Therefore, it is difficult to evaluate the pure added value of kinetic information compaed to the morphological analysis alone in the designed study. We used the suggested interpretation guideline of AB-MRI for objective morphological analysis alone, and no additional information was considered, although the guidelines actually recommend considering kinetic information if available. This study design might explain the low specificity of our study results compared with that of other reports on the performance of AB-MRI. In our study, the sensitivity and specificity of AB-MRI with morphological analysis alone were 94.9% and 29.7% for the detection of all malignancy and 94.6% and 26.5% for the detection of invasive cancer, respectively. Although the sensitivities were similar, the specificities were far lower than previous reports, with pooled values ranging from 81.8 to 100% and from 75.4 to 97.2%, respectively [21]. 

We set an optimal cutoff value that did not sacrifice sensitivity while significantly increasing specificity. Therefore, we focused on downgrading lesions that did not require subsequent biopsy. Improving the specificity has significant clinical implications, as the biopsy of many benign lesions places a high emotional burden on women and adds substantial additional costs, reducing its feasibility as a screening tool [25,26,27]. The use of quantitative kinetic information derived from AB-MRI may reduce the time and cost burdens by reducing false-positive and recall rates, as unnecessary biopsy could have been avoided in 34 benign lesions in our results if lesions with an enhancement degree <90% were considered benign. However, based on these criteria, three malignant lesions could have been missed, two of which were invasive. One IDC was a tumor that recurred at the previous operation site; the other was an increased mass compared to the previous MRI. Therefore, in addition to considering the kinetic information, the consideration of situations such as a newly developed mass compared to previous imaging studies is needed to maintain sensitivity. 

In our study, we derived the enhancement curve type from two post-contrast images obtained 60 s and 120 s after contrast injection. Although few studies have reported AB-MRI with two post-contrast sequences, they showed that the diagnostic performance of the shortened protocol was similar to that for FDP-MRI, with a reduced scan time [28]. Our study results showed that kinetic curve type derived from first and second post-contrast images differed significantly between benign and malignant lesions, with malignant lesions showing more frequent washout type and benign lesions showing more persistent enhancement, suggesting that early kinetic information from only two post-contrast images at 60 s and 120 s was sufficient to assess the enhancement curve type of the lesion. However, there was no added value as this significantly decreased sensitivity at the cost of a significantly increased specificity. Since our results concluded that first post-contrast images are sufficient for differential diagnosis, we support the use of the currently more widely used AB-MRI protocol, which obtains only one post-contrast image.

Many studies have evaluated the kinetics of MRI, focusing on sequences with a temporal resolution of <10–20 s, such as ultrafast imaging [29,30,31]. However, these methods require additional scanning with a high-end MRI equipment. The main advantage of our results is that they can be quickly incorporated into current clinical practice by using commercially available CAD, without obtaining additional sequences. 

We obtained consistent results with both 1.5-T and 3.0-T; thus, our results could be applied widely, regardless of the type of MRI magnetic field strengths. However, the increase in the specificity of combined diagnostic criteria of morphology and enhancement degree was higher at 1.5-T than at 3.0-T. As 3.0-T offers a higher signal-to-noise ratio, better image quality, and diagnostic confidence than 1.5-T, using CAD-assisted enhancement degree information might be more helpful for differential diagnosis at 1.5-T [32,33,34].

This study has several limitations. First, this was a retrospective study in an enriched cohort of patients who underwent breast cancer surgery and included only lesions with BI-RADS category 3, 4, and 5. As we did not include MRI with negative results, the absolute values of diagnostic performance might not be representative and not reflect the realities of clinical practice. However, we intended to minimize the interobserver variability of morphological analysis by using the interpretation guidelines and focused on the added value of kinetic information. Second, because we manually identified a single voxel in the area of highest enhancement within the lesions that could not be detected by CAD, t hismight not have represented the characteristics of the entire lesion, as malignant masses are more heterogeneous than benign masses [35]. However, the BI-RADS guidelines suggest analyzing the lesion’s most suspicious portion to assess kinetic information. Thus, we considered the area of highest enhancement on the first post-contrast-enhanced image to be the most suspicious portion of a lesion. Third, the sizes of benign and malignant lesions differed significantly, with malignant lesions being larger in diameter. This might be because some cases of multiple stippled enhancing nodules that could be considered as BPE were categorized as BI-RADS category 3 and included in our study. However, even if the lesion is small in size, it is less likely to affect the enhancement degree because we analyzed the quantitative voxel value of the enhancement degree of the most suspicious portion of the lesion.

In conclusion, we suggest a method to best utilize the kinetic information provided by the abbreviated protocol, as follows. The combination of the enhancement degree on the first post-contrast-enhanced image (≥90% for all malignancy and ≥107% for invasive cancer) with morphological analysis can improve the specificity of AB-MRI in differentiating malignant and benign breast lesions without compromising sensitivity. We suggest that a single post-contrast acquisition is sufficient, as additional post-contrast imaging to observe kinetic curve type did not further improve the differential diagnosis over the simple assessment of the degree of enhancement.

## Figures and Tables

**Table 1:** Patient and lesion characteristics of benign and malignant lesions.

Characteristics	Benign (*n* = 148)	Malignant (*n* = 59)	*p* Value
Age (years) *	49.1 ± 9.1	49.7 ± 7.9	0.561
Family history of breast cancer			0.498
No	135 (91.2)	52 (88.1)	
Yes	13 (8.8)	7 (11.9)	
*BRCA1* or *BRCA2* mutation			0.225
Negative	130 (87.8)	48 (81.4)	
Positive	18 (12.2)	11 (18.6)	
Tumor size (cm) †	0.7 (0.3-10)	1.3 (0.3-6.2)	<0.001
Lesion type			0.040
Mass	122 (82.4)	41 (69.5)	
NME	26 (17.6)	18 (30.5)	
Mass margin			<0.001
Circumscribed	64 (52.5)	5 (12.2)	
Not circumscribed	58 (47.5)	36 (87.8)	
Mass internal enhancement			0.012
Homogeneous	55 (45.1)	9 (22.0)	
Heterogeneous	67 (54.9)	32 (78.0)	
Mass rim enhancement			0.014
Yes	10 (8.2)	7 (17.1)	
No	112 (91.8)	34 (82.9)	
NME distribution			0.400
Linear/segmental	14 (53.8)	12 (66.7)	
Focal/regional/multiple regions/diffuse	12 (46.2)	6 (33.3)	
NME internal enhancement			0.790
Homogeneous	1 (3.8)	1 (5.6)	
Heterogeneous/clumped/clustered ring	25 (96.2)	17 (94.4)	
Enhancement degree (%) *	141.3 ± 97.8	238.0 ± 128.5	<0.001
Enhancement rate			<0.001
Slow	24 (16.2)	0 (0)	
Intermediate	35 (23.7)	5 (8.5)	
Rapid	89 (60.1)	54 (91.5)	
Enhancement curve type			<0.001
Persistent	100 (67.6)	18 (30.5)	
Plateau	31 (21.0)	14 (23.7)	
Washout	17 (11.5)	27 (45.8)	
BI-RADS category			<0.001
3 (Probably benign)	114 (77.0)	9 (15.3)	
4A (Low suspicion for malignancy)	27 (18.2)	13 (22.0)	
4B (Moderate suspicion for malignancy)	7 (4.7)	13 (22.0)	
4C (High suspicion for malignancy)	0 (0)	12 (20.3)	
5 (Highly suggestive of malignancy)	0 (0)	12 (20.3)	
Biopsy recommend by guideline			<0.001
No	44 (29.7)	3 (5.1)	
Yes	104 (70.3)	56 (94.9)	
MRI magnetic field strength			0.420
1.5-T	46 (31.1)	15 (25.4)	
3.0-T	102 (68.9)	44 (74.6)	
Screening round			0.490
First	98 (66.2)	42 (71.2)	
Second or more	50 (33.8)	17 (28.8)	

Unless otherwise specified, data are number of lesions with percentage in parentheses. * Number is mean ± standard deviation. † Number is median with ranges in parentheses. BI-RADS = Breast Imaging Reporting and Data System; BRCA = BReast CAncer gene; MRI = magnetic resonance imaging; NME = non-mass enhancement.

**Table 2:** Receiver operating characteristics curve analysis of parameters for differentiating benign and malignant breast lesions.

Parameter	AUC	95% CI	*p* Value	Cutoff
Morphological analysis alone	0.62	0.58–0.67		
Enhancement degree (%)				
All malignancy	0.74	0.67–0.81	0.009	90%
Invasive cancer	0.72	0.64–0.81	0.029	107%
Enhancement rate				
All malignancy	0.66	0.61–0.72	0.265	
Invasive cancer	0.65	0.59–0.70	0.304	
Enhancement curve type				
All malignancy	0.72	0.65–0.80	0.036	Plateau
Invasive cancer	0.71	0.62–0.80	0.066	
Tumor size (cm)				
All malignancy	0.73	0.65–0.81	0.012	0.5 cm
Invasive cancer	0.72	0.63–0.82	0.018	0.6 cm

AUC = area under curve; CI = confidence interval.

**Table 3:** Diagnostic performance of parameters for differentiating benign and malignant breast lesions.

Parameter	Sensitivity (%)	*p* Value	Specificity (%)	*p* Value
For detection of all malignancy				
Morphological analysis alone	94.9		29.7	
Morphological analysis + Enhancement degree ≥ 90%				
All	89.8	0.080	52.7	<0.001
1.5-T	73.3	0.083	76.1	<0.001
3.0-T	95.5	N/A	42.2	<0.001
Morphological analysis + Enhancement curve type ≥ plateau	66.1	<0.001	79.1	<0.001
Morphological analysis + Enhancement degree ≥ 90% + Enhancement curve type ≥ plateau	64.4	<0.001	80.4	<0.001
For detection of invasive cancer				
Morphological analysis alone	94.6		26.5	
Morphological analysis + Enhancement degree ≥ 107%				
All	86.5	0.083	57.6	<0.001
1.5-T	70.0	0.083	80.4	<0.001
3.0-T	92.6	N/A	47.9	<0.001
Morphological analysis + Size ≥ 0.6 cm				
All	86.5	0.083	38.8	<0.001
1.5-T	90.0	0.371	41.2	0.083
3.0-T	85.2	0.157	37.8	<0.001
Morphological analysis + Enhancement degree ≥ 107% + Size ≥ 0.6 cm	78.4	0.014	63.5	<0.001

## Data Availability

The datasets generated or analyzed during the study are available from the corresponding author on reasonable request.

## Supplementary Materials

The following supporting information can be downloaded at: https://www.mdpi.com/article/10.3390/diagnostics13010136/s1, Table S1: Abbreviated protocol of screening breast MRI. Figure S1: Interpretation guideline of mass on AB-MRI. Figure S2: Interpretation guideline of non-mass enhancement on AB-MRI.
