# Correction: Kim et al. Assessment of Enhancement Kinetics Improves the Specificity of Abbreviated Breast MRI: Performance in an Enriched Cohort. *Diagnostics* 2023, *13*, 136

**DOI:** 10.3390/diagnostics13101713

**Published:** 2023-05-12

**Authors:** Haejung Kim, Eun Young Ko, Ka Eun Kim, Myoung Kyoung Kim, Ji Soo Choi, Eun Sook Ko, Boo-Kyung Han

**Affiliations:** Department of Radiology and Center for Imaging Science, Samsung Medical Center, Sungkyunkwan University School of Medicine, Seoul 06351, Republic of Korea

There were errors in the original publication [1].

The CAD system version and an inequality sign in the definition should be corrected.

A correction has been made to 2. Materials and Methods, 2.4. Kinetic Analysis, 1st paragraph:

The sentence “Kinetic information was retrospectively analyzed on a dedicated workstation using a CAD system (CadstreamTM version 4.1.3, Merge Healthcare, Inc., Hartland, WI, USA).” should be replaced with “Kinetic information was retrospectively analyzed on a dedicated workstation using a CAD system (CadstreamTM v6.0, Merge Healthcare, Inc., Hartland, WI, USA).”.

The sentence “The enhancement rate, defined as the signal change between the pre- and first post-contrast images, was categorized as slow (<50% increase), medium (50–100%), or rapid (≥100%).” should be replaced with “The enhancement rate, defined as the signal change between the pre- and first post-contrast images, was categorized as slow (<50% increase), medium (50–100%), or rapid (>100%).”.

A *p*-value should be corrected.

A correction has been made to 3. Results, 3.1. Patient and Lesion Characteristics, 1st paragraph:

The sentence “In the morphological analysis, malignant masses more frequently showed non-circumscribed margins (*p* < 0.001), heterogeneous enhancement (*p* = 0.012), and rim enhancement (*p* = 0.012).” should be replaced with “In the morphological analysis, malignant masses more frequently showed non-circumscribed margins (*p* < 0.001), heterogeneous enhancement (*p* = 0.012), and rim enhancement (*p* = 0.014).”.

Inequality signs and an AUC value should be corrected. 

A correction has been made to 3. Results, 3.2. Receiver Operating Characteristics Curve Analysis of Parameters for Differentiating Benign and Malignant Breast Lesions, 1st paragraph:

The sentence “For the detection of all malignancies including in situ carcinoma, the enhancement degree, enhancement curve type, and size showed significantly better AUC values compared to morphological analysis alone (0.72–0.74 vs. 0.62; *p* ≤ 0.05 for all parameters).“ should be replaced with “For the detection of all malignancies including in situ carcinoma, the enhancement degree, enhancement curve type, and size showed significantly better AUC values compared to morphological analysis alone (0.72–0.74 vs. 0.62; *p* < 0.05 for all parameters).”.

The sentence “For the detection of invasive cancers, enhancement degree and size showed significantly better AUC values compared to morphological analysis alone (0.72 for each vs. 0.6; *p* ≤ 0.05 for both parameters).” should be replaced with “For the detection of invasive cancers, enhancement degree and size showed significantly better AUC values compared to morphological analysis alone (0.72 for each vs. 0.61; *p* < 0.05 for both parameters).”.

Incorrect dividing lines inside the Table 1 and Table 3 and the indentations should be corrected. The corrected Table 1 and Table 3 appear below. 

In the fifth line of Figure S1, “circumscribed” should be corrected to “heterogeneous enhancement.” The corrected Figure S1 appears below. 

The authors state that the scientific conclusions are unaffected. This correction was approved by the academic editor. The original publication has also been updated.

## Figures and Tables

**Figure S1:**
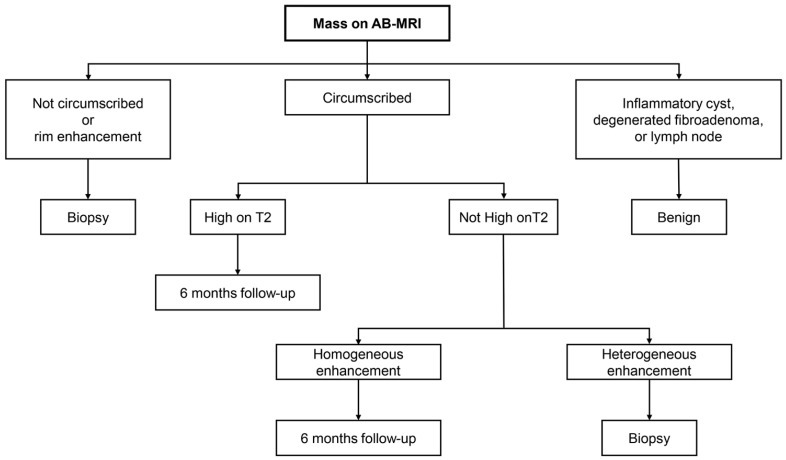
Interpretation guideline of mass on AB-MRI.

**Table 1:** Patient and lesion characteristics of benign and malignant lesions.

Characteristics	Benign (*n* = 148)	Malignant (*n* = 59)	*p* Value
Age (years) *	49.1 ± 9.1	49.7 ± 7.9	0.561
Family history of breast cancer			0.498
No	135 (91.2)	52 (88.1)	
Yes	13 (8.8)	7 (11.9)	
*BRCA1* or *BRCA2* mutation			0.225
Negative	130 (87.8)	48 (81.4)	
Positive	18 (12.2)	11 (18.6)	
Tumor size (cm) †	0.7 (0.3–10)	1.3 (0.3–6.2)	<0.001
Lesion type			0.040
Mass	122 (82.4)	41 (69.5)	
NME	26 (17.6)	18 (30.5)	
Mass margin			<0.001
Circumscribed	64 (52.5)	5 (12.2)	
Not circumscribed	58 (47.5)	36 (87.8)	
Mass internal enhancement			0.012
Homogeneous	55 (45.1)	9 (22.0)	
Heterogeneous	67 (54.9)	32 (78.0)	
Mass rim enhancement			0.014
Yes	10 (8.2)	7 (17.1)	
No	112 (91.8)	34 (82.9)	
NME distribution			0.400
Linear/segmental	14 (53.8)	12 (66.7)	
Focal/regional/multiple regions/diffuse	12 (46.2)	6 (33.3)	
NME internal enhancement			0.790
Homogeneous	1 (3.8)	1 (5.6)	
Heterogeneous/clumped/clustered ring	25 (96.2)	17 (94.4)	
Enhancement degree (%) *	141.3 ± 97.8	238.0 ± 128.5	<0.001
Enhancement rate			<0.001
Slow	24 (16.2)	0 (0)	
Intermediate	35 (23.7)	5 (8.5)	
Rapid	89 (60.1)	54 (91.5)	
Enhancement curve type			<0.001
Persistent	100 (67.6)	18 (30.5)	
Plateau	31 (21.0)	14 (23.7)	
Washout	17 (11.5)	27 (45.8)	
BI-RADS category			<0.001
3 (Probably benign)	114 (77.0)	9 (15.3)	
4A (Low suspicion for malignancy)	27 (18.2)	13 (22.0)	
4B (Moderate suspicion for malignancy)	7 (4.7)	13 (22.0)	
4C (High suspicion for malignancy)	0 (0)	12 (20.3)	
5 (Highly suggestive of malignancy)	0 (0)	12 (20.3)	
Biopsy recommend by guideline			<0.001
No	44 (29.7)	3 (5.1)	
Yes	104 (70.3)	56 (94.9)	
MRI magnetic field strength			0.420
1.5-T	46 (31.1)	15 (25.4)	
3.0-T	102 (68.9)	44 (74.6)	
Screening round			0.490
First	98 (66.2)	42 (71.2)	
Second or more	50 (33.8)	17 (28.8)	

Unless otherwise specified, data are number of lesions with percentage in parentheses. * Number is mean ± standard deviation. NME = non-mass enhancement † Number is median with ranges in parentheses. BI-RADS = Breast Imaging Reporting and Data System; BRCA = BReast CAncer gene; MRI = magnetic resonance imaging.

**Table 3:** Diagnostic performance of parameters for differentiating benign and malignant breast lesions.

Parameter	Sensitivity (%)	*p* Value	Specificity (%)	*p* Value
For detection of all malignancy				
Morphological analysis alone	94.9		29.7	
Morphological analysis + Enhancement degree ≥ 90%				
All	89.8	0.080	52.7	<0.001
1.5-T	73.3	0.083	76.1	<0.001
3.0-T	95.5	N/A	42.2	<0.001
Morphological analysis + Enhancement curve type ≥ plateau	66.1	<0.001	79.1	<0.001
Morphological analysis + Enhancement degree ≥ 90% + Enhancement curve type ≥ plateau	64.4	<0.001	80.4	<0.001
For detection of invasive cancer				
Morphological analysis alone	94.6		26.5	
Morphological analysis + Enhancement degree ≥ 107%				
All	86.5	0.083	57.6	<0.001
1.5-T	70.0	0.083	80.4	<0.001
3.0-T	92.6	N/A	47.9	<0.001
Morphological analysis + Size ≥ 0.6 cm				
All	86.5	0.083	38.8	<0.001
1.5-T	90.0	0.371	41.2	0.083
3.0-T	85.2	0.157	37.8	<0.001
Morphological analysis + Enhancement degree ≥ 107% + Size ≥ 0.6 cm	78.4	0.014	63.5	<0.001
