# Supplementary material for: Assessment of Enhancement Kinetics Improves the Specificity of Abbreviated Breast MRI: Performance in an Enriched Cohort
Source: Diagnostics (Basel). 2022 Dec 30;13(1):136. doi: 10.3390/diagnostics13010136 (PMC9818206; doi:10.3390/diagnostics13010136)
Supplement: Supplementary file 1 [file diagnostics-13-00136-s001.zip › diagnostics-2076401-supplementary.pdf]

Supplementary Table

Table S1. Abbreviated protocol of screening breast MRI.

**Scanning**	**Series of images**	**Plane**
T2 without fat suppression	T2 without fat suppression	Axial
T1 contrast-enhanced 3D dynamic with fat suppression	3D dynamic contrast-enhanced	Axial
Pre-contrast	Pre-contrast	
Post-contrast first (60 sec)	Post-contrast first	
Post-contrast second (120 sec)	Post-contrast second	
	Standard subtraction (first-pre, second-pre)	Axial
	Reversed subtraction (first-second)	Axial
	MIP, both breasts	Axial, Sagittal

3D = three-dimensional, MIP = maximum intensity projection.

Supplementary Figures

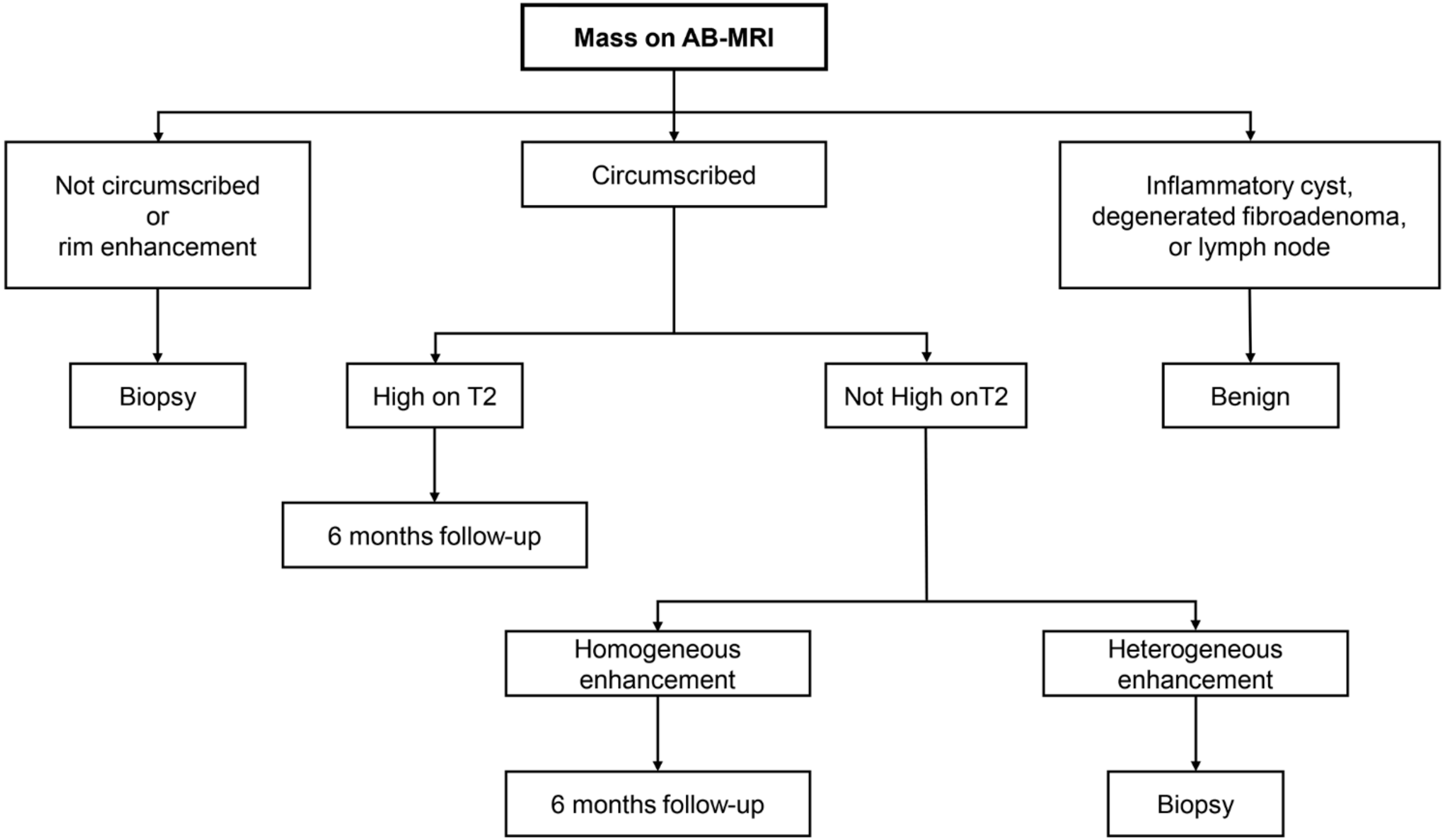

Figure S1. Interpretation guideline of mass on AB-MRI.

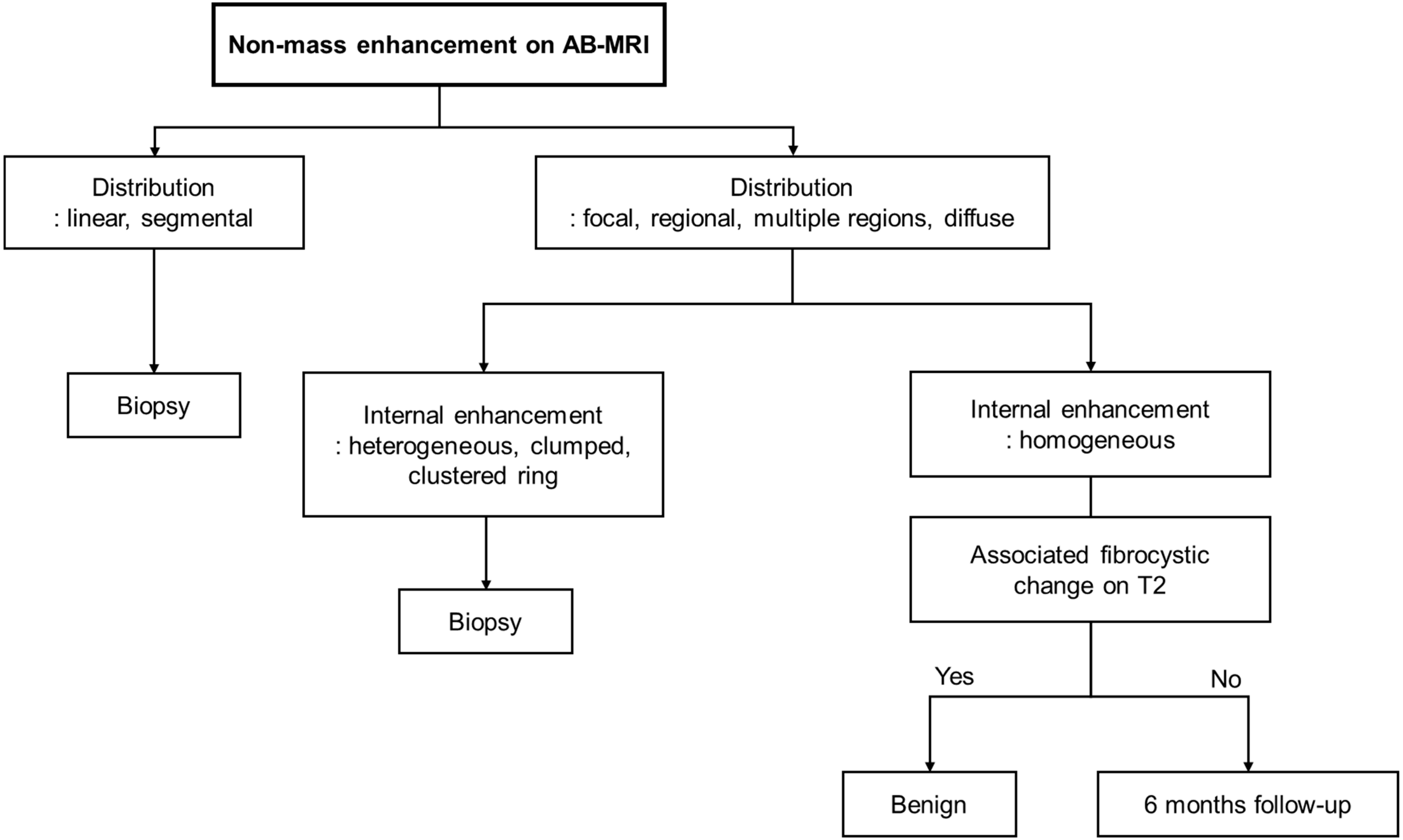

Figure S2. Interpretation guideline of non-mass enhancement on AB-MRI.
